# Silicon Improves Yield Performance by Enhancement in Physiological Responses, Crop Imagery, and Leaf and Culm Sheath Morphology in New Rice Line, PadiU Putra

**DOI:** 10.1155/2021/6679787

**Published:** 2021-05-31

**Authors:** Zulkarami Berahim, Mohamad Husni Omar, Nurul-Idayu Zakaria, Mohd Razi Ismail, Rhushalshafira Rosle, Nor Athirah Roslin, Nik Norasma Che'Ya

**Affiliations:** ^1^Institute of Tropical Agriculture and Food Security, Universiti Putra Malaysia, 43400 Serdang, Selangor, Malaysia; ^2^Department of Crop Science, Faculty of Agriculture, Universiti Putra Malaysia, 43400 Serdang, Selangor, Malaysia; ^3^Department of Agriculture Technology, Faculty of Agriculture, Universiti Putra Malaysia, 43400 Serdang, Selangor, Malaysia

## Abstract

The PadiU Putra rice line is a blast-resistant and high-yield rice line with high potential. The application of topdressing and the foliar applied method of silicon (Si) treatments could strengthen the culm to resist breakage and ultimately increase yield production. Treatments which consisted of a control, a Si topdressing, and a Si foliar applied were arranged in a randomised complete block design. At 55 days after transplanting (DAT), the foliar applied Si treatments had 59% higher dry matter partitioning to the roots. Meanwhile, at 75 DAT, both Si foliar applied and topdressing method showed increased assimilate partitioning into the culm sheath by 29% and 49%, respectively. Dark green and light yellowish colours were obtained in both Si treatments using UAV, indicating similar results to physiological responses. Remarkably, Si foliar applied treatments enhanced the diameter and width of the outer and inner layers of the diameter of vascular bundles at 75 DAT by 58, 181, and 80%, respectively. The yield production of rice increased by 53% in the Si foliar applied, compared to the control, and produced a 1.63 benefit-cost ratio.

## 1. Introduction

Rice, *Oryza sativa* L., is a major cereal crop. Sixty per cent of the global population (4.5 billion) lives in Asia, a figure which will increase to 9.3 billion in the year 2050. The demand for rice will also increase by up to 50% due to the increasing population [1]. In Malaysia, rice is the third most widely planted crop after oil palm and rubber. In 2013, 674,332 hectares (ha) of land was used for plantations with an average yield of only 4.5 t ha^−1^ and with a self-sufficiency level (SSL) of 72% [2]. Following that, the Malaysian government has further targeted an increase in the average paddy yield from 4.5 t ha^−1^ to 6.0 t ha^−1^ [3]. Recently, a new variety of rice, currently called PadiU Putra, was developed by [4, 5], in which resistance genes from the rice variety Pongsu Seribu 2 were pyramided into MR219. This new variety is claimed to be a blast-resistant rice variety with high yield potential and is to be released for commercial cultivation [5]. As a new variety, it is also exposed to the lodging problem due to being a taller plant. Lodging is defined as the permanent displacement of a stem from its upright position [6]. It mostly occurs just before harvest when the plant, in particular the lower portion of the stem, is unable to withstand the weight of the panicle. To date, three types of lodging have been recorded, namely, bending of stems at the base, breakage of stems at any point along the length, and root lodging [7].

The lodging resistance of a rice plant could be achieved through a suitable plant height and shorter basal stems [8]. However, the study conducted by [9] showed that the application of silicon (Si) fertiliser increased the plant height. Leaf and culm morphology can be affected by the application of Si fertiliser; however, few studies have focused on the structural changes to the leaf and culm. The present study and [10–12] provide details on variations in the silica cell structure and Si deposition process by using scanning electron microscopy (SEM). SEM provides Si localisation in various parts of the rice plant such as the leaf-blade, leaf-sheath, stem, root, and husk. At the same time, the composition and structure of plant cell walls are ideally suited to the functions they perform [11]. The plant cell wall provides mechanical support to cells, tissues, and the entire plant body against lodging that might occur [13]. Sclerenchyma cells, which have both primary and thick secondary walls, provide the major mechanical support in the mature regions of the plant body [11].

Si is one of the main elements in the Earth's crust, second to oxygen in abundance [14]. Si is primarily present in the epidermal cells which provide structural rigidity to the plant. A remarkable unique feature of Si is that once it is deposited as silica gel, it is not redistributed to other parts of the plant [15]. Si accumulation sometimes exceeds that of crucial plant nutrients, especially in the grass family [16]. Rice is known as a Si accumulator [17], and the accumulation of Si in leaves and tissues can improve rice growth as a result of increasing the photosynthetic rate [18]. It can also enhance biomass partitioning, improve grain quality [9, 19], and increase yield [20]. Si is likely deposited in the cell walls of shoots, the intercellular regions, and silica cells [21]. A mature silica cell with observable Si deposition is referred to as a silica body. Si is deposited in two types of silica cells in the rice leaf to form two corresponding silica bodies, one dumbbell-shaped and the other bulliform-shaped [21]. The number of silica bodies in shoots correlates positively with the Si content in shoots. Si is also considered an environmentally friendly element in relation to soils, fertilisers, and plant nutrition [22].

The method of fertiliser application is essential as it affects nutrient accessibility. Si was commonly applied as a base fertiliser [9] or foliage fertiliser [23]. Plants absorb Si most effectively when it is applied at the tillering to booting stages [24]. Interestingly, these methods are encouraging for nutrients that are taken up by means of vascular transportation. On the other hand, foliar is relatively easy to apply at manageable rates. However, the topdressing method for solid fertiliser could be supplied at higher rates [10]. In addition, these methods are favourable for nutrients that are taken up by means of vascular transportation. Moreover, the ease of application with Si could also make this form of application more viable and economical [10].

The use of unmanned aerial vehicles (UAVs) for precision agriculture has been increasing, and these machines have been designed specifically to ease problematic agricultural activities [25]. Initially, UAVs were used to take aerial images of a farm [26]. A key advantage of using UAV is that this allows farmers to monitor and obtain valuable information about the field and crop conditions such as crop health, vigour, and growth stages and ultimately to determine yield [27].

Thus, the main objective of this experiment was to examine the topdressing and foliar forms of Si applications on the physiological performance and structural changes of leaf and culm sheaths to enable an evaluation of the yield improvement and a cost analysis of the PadiU Putra rice line.

## 2. Materials and Methods

### 2.1. Field Experiment and Soil Properties

The field experiment was conducted in major rice granary areas from October 2018 until February 2019. The location of the study was at Kampung Maharaja, Tunjang, Kubang Pasu, Jitra, and Kedah under the Muda Agricultural Development Authority (MADA)(6° 16′ N 100° 21′ E, 76 m elevation). The soil has a clay loam texture (22.9% sand, 49.1% silt, and 28% clay) with pH 5.3 and was used as the cultivation medium. The soil nutrient status was 0.38% total N, 43 mg kg^−1^available P, 167 mg kg^−1^ available K, 1559 mg kg^−1^ available Ca, 433 mg kg^−1^ available Mg, 529 mg kg^−1^ available Fe, 23 mg kg^−1^ available Mn, 3.7 mg kg^−1^ available Zn, and 1.1 mg kg^−1^ available Cu.

### 2.2. Planting Materials and Plant Establishment

A transplanted blast-resistant and high-yielding rice line with high potential (MR219 X Pongsu Seribu 1 through SSR markers), known as PadiU Putra, was used in the experiment. The field was thoroughly prepared and levelled using a leveller machine before transplanting took place. Twenty-day-old seedlings were transplanted at 20 × 15 cm spacing. Nine plots for each experiment with 1,637 m^2^ (24.4 × 67.1 m) areas were prepared and separated by a 0.5 m barrier. Each plot was fertilised with NPK compound fertiliser at 360 kg ha^−1^, 175 kg ha^−1^, and 175 kg ha^−1^ at 15, 50, and 70 days after transplanting (DAT). Urea was top-dressed at a rate of 100 kg ha^−1^ at 35 DAT. In each plot, a uniform plant stand was maintained, and standard agronomic practices were followed, as recommended by government agency practices.

### 2.3. Experimental Design and Statistical Analysis

In this experiment, treatments comprised two sources of methods of Si application (control treatments/untreated: without any additional Si source; topdressing treatments: plants supplied with Si in tablet form; and foliar applied treatments: plants supplied with Si through the foliar spray method) were arranged in a randomised complete block design (RCBD) with three replications. Si tablets were supplied by the Behn Meyer AgriCare (M) Company, Malaysia, at a rate of 3 kg ha^−1^ at 45 and 65 DAT, corresponding to the vegetative and reproductive stages, respectively. Two thousand and five hundred parts per million of Si foliar application with 0.01% dimethyl sulfoxide (DMSO) as an adjuvant was performed by spraying plants uniformly to the point of run-off (approximately 140 L ha^−1^) using a Stihl type mist blower (Stihl Sdn Bhd, Malaysia, 14 L) with a constant flow. The treatments were applied between 9:00 to 11:00 am on a day with clear sky at 45 and 65 DAT. Water was maintained at 5 cm by irrigation throughout the rice cultivation periods, and a 0.5 m boundary was prepared to avoid assortment. The Statistical Analysis System (SAS 9.2) by least significant different (LSD) at *P* ≤ 0.05 was performed.

### 2.4. Growth Measurements

#### 2.4.1. Plant Height and Tiller Number

The plant height was measured according to the methods described by [28], where the measurement was made from the plant base to the tip of the highest leaf blade. The tiller number per square metre was counted by a fully expanded tiller. Samples with three replications were taken after ten days of Si treatment had been applied to the plants using all parameters. The purpose of this was to allow enough time for the plants to respond to the treatments. At the same time, two critical times in the rice growth stages were involved in this sampling time, the vegetative and reproductive stages. Four samples per square metre for each treatment were taken randomly and counted at 55 and 75 DAT.

#### 2.4.2. Average Diameter of Internodes and Diameter of Internodes at 20 cm

The average diameter of the internodes was measured at each internode midregion using a digital Vernier caliper from three representative plants from each treatment, with three replications, at 55 and 75 DAT. The diameter of the internodes at 20 cm was measured at the section of the internodes at 18 to 22 cm from the basal.

### 2.5. Photosynthesis, Stomatal Conductance, and Chlorophyll Content

The photosynthesis rate was measured on fully expanded young leaves (the third leaf from the top) at 0900-1100 am on a day with clear sky using a portable photosynthesis system (Li-6400XT, LI-COR, Lincoln, Nebraska, USA). The measurements were taken on the abaxial surface at a CO_2_ reference rate of 400 *μ*mol m^−2^ s^−1^ at 55 and 75 DAT. The photosynthetic photon flux density (PPFD) was 900 mmol m^−2^ s^−1^. The stomatal conductance was derived from the same photosynthesis measurements described earlier. The chlorophyll content of the leaves was measured by the indirect method using a Portable Minolta SPAD 502 Plus chlorophyll meter (Delta T, UK). The third fully expanded leaf from the top was chosen for data measurements at 55 and 75 DAT. Three replications were taken for each of the parameters.

### 2.6. Biomass Partitioning

Three plants were harvested from each treatment at 55 and 75 DAT. They were partitioned into roots, culms, and leaves to determine the dry weights of each part. The dry weights of the plant parts were measured using a digital balance (QC35EDE-S Sartorius, Germany) after drying them in an oven at 72°C for three days until the weight became constant. The total biomass was calculated based on the total dry weights of the leaf, culm, root, and total yield. The root to shoot ratio was calculated using the following formula [29]: Root : Shoot Ratio = Total Root Dry Weight/Total Shoot Dry Weight.

### 2.7. Rice Crop Monitoring Using a Multirotor Unmanned Aerial Vehicle (UAV) and Red, Green, and (RGB) Digital Camera

For aerial imaging, a multirotor UAV DJI Phantom 4 Pro V2.0 with a gimbal-stabilised 4 K60 and 20 megapixel RGB digital camera attached was used to fly above the experimental field area. The flight plan was designed before the data acquisition by using the *DroneDeploy* software on a tablet. The altitude for the data acquisition of RGB images was set at 80 m, equivalent to a 2.19 cm spatial resolution. To avoid biased colours and lighting due to cloud shadows, the data collection was conducted in the morning under clear skies and at low wind speed conditions between 08:30 am and 11:00 am local time at 55 and 75 DAT. The camera settings were adjusted according to the light conditions and set to a fixed exposure for each flight.

### 2.8. Image Processing

Agisoft Metashape Professional software (http://www.agisoft.com/) was used to develop and align the imagery mosaic using Structure from Motion (SfM) algorithms. For each set of images, Agisoft PhotoScan software aligned the images and built point cloud models of the surface. Agisoft allows the generation and visualisation of a dense point cloud model, based on the estimated camera positions, to combine into a single dense point cloud [30]. The software provides a user-friendly process for mosaicking the imagery. The imagery was added and aligned using the Align Photo function. Then, the imagery generated and visualised a dense point cloud model based on the estimated camera position using the Build Dense Cloud function. It calculated the depth information for each camera, which could be combined into a single dense point cloud [30]. The geometrics of the map were reconstructed due to the poor texture of some elements of the scene and noisy or poorly focused images (known as outliers among the points) by using the Build Mesh function. The images were used to build the texture exported as a mosaicked orthophoto image [30]. Finally, the mosaicked orthophoto generated a Digital Surface Model (DSM), and the DSM and orthoimage were imported to build the 2.5 digital models.

### 2.9. Scanning Electron Microscopy Analysis of Leaf and Culm

At 55 and 75 DAT, the middle section of the flag leaves (approximately 0.5 cm in width) and the section of internodes at 18 to 22 cm from the basal (approximately 0.3 cm in width) from each treatment were cut with a sharp blade and fixed in fixative (4% glutaraldehyde) for 2 days at 4°C. The samples were washed in a buffer (0.1 M sodium cacodylate) with three changes of 30 minutes each. The samples were postfixed in osmium tetraoxide for 2 hours at 4°C and were washed again in the buffer (0.1 M sodium cacodylate) with three changes of 30 minutes each. The samples were then dehydrated in each graded acetone series of 35, 50, 75, and 95% (30 minutes) and 100% (1 hour each for three changes) followed by critical point drying (Baltec CPD 030). The leaves and culm specimens were mounted on aluminium stubs, covered with double-sided adhesive tape, and sputter-coated with gold with a sputter coater (Baltec SCD 005). They were viewed under a scanning electron microscope (JEOL JSM 6400, Japan) at an accelerating voltage of 15 kV. Three replications were taken for each of the parameters.

### 2.10. Yield Components, NPV, and BCR

After 80% grain maturity, all plants were harvested from each plot of treatments in the field trials experiment. Sampling was carried out by harvesting plants at maturity within a quadrant of 1 m^2^ to determine the yield components. The panicle number of each collected plant was counted to calculate the total number of panicles from the unit area (1 m^2^). Panicle per hill, grain number per panicle, and percentage of filled grains per panicle were counted and calculated manually. The thousand-grain weight (g) was also obtained using an electronic balance (QC 35EDE-S Sartorius, Germany). Ten panicle-bearing tillers from each treatment were sampled. Prior to weighing the grains, fully filled grains were manually separated from the unfilled grains. The percentage of filled grains per panicle was derived from the ratio of the number of fully ripened grains (filled grains) to the total number of grains per panicle per average hill (Yoshida 1981).

The yield was determined with three replications for each treatment at the end of the experiment at 120 DAT. The yield was separately harvested by a harvester machine (World Star, WS70 Plus, China), loaded into separate lorries, and weighed in the rice mills (BERNAS Sdn Bhd). The yield of rice divided by the area in hectares, noted on the purchasing receipt, was collected from the rice mills. Moreover, the Malaysian government gives an additional incentive for every metric ton of rice yield increase, compared with the usual yield per hectare [31]. To determine the economic feasibility of both methods of Si application, a benefit-cost analysis and a net present revenue were carried out. The benefit-cost ratio (BCR) was calculated using the method of [32], whereas the net present value (NPV) was determined as described by [33].

(1) $$\begin{array}{c} \text{The benefit}-\text{cost ratio}\left(\text{BCR}\right)=\text{TCTR},\text{Net present value}\left(\text{NPV}\right)=\Sigma \left(\text{TR}-\text{TC}\right)t\left(1+r\right)\text{tnt}=1, \end{array}$$

where TC is the total cost, TR is the total revenue, and *r* is the discount rate of rice field for per season (*t*). If NR or NPV > 0, then the total revenue is greater than the total cost; if NR or NPV = 0, then the total revenue is equal to the total cost; and if the NR or NPV < 0, then the total revenue is less than the total cost. In this study, NR and NPV are measured in Malaysian Ringgit (RM) and are based on one hectare. If BCR > 1, then the total revenue is greater than the total cost; if BCR = 1, then the gross revenue is equal to the total cost; and if the BCR < 1, then the gross revenue is less than the total cost.

## 3. Results

### 3.1. Plant Growth Parameters

#### 3.1.1. Plant Height and Tiller Number

The plant height and tiller number per square metre for different Si method applications at 55 and 75 DAT in rice plants are shown in Table 1. The results showed that the use of Si foliar applied and topdressing treatments showed significantly reduced plant height by 17 and 13% at 55 DAT, respectively, compared to the control. The results further revealed that both methods of Si treatments imposed caused slight differences in plant height during the reproductive stages of the rice plant (75 DAT). According to the results, the tiller number of both methods of Si treatments was not statistically different from the control at both DAT.

#### 3.1.2. Diameter and Length of Internodes

The diameter of internodes was not statistically significant among treatments at 55 DAT (as shown in Table 2). The diameter of internodes was significantly different at 75 DAT in the control, followed by the Si foliar applied and topdressing treatments, with 4.9, 4.0, and 3.5 mm, respectively. On the other hand, the length of internodes for Si topdressing treatments was significantly different, by 22%, at 55 DAT compared to the other treatments. The results revealed a reduction in the internode length at 75 DAT in both methods of Si treatments. Compared to the control, the reductions were by 14% in the topdressing and 9% in the foliar applied treatments.

### 3.2. Physiological Responses

The photosynthesis rate (Pn) on different Si method applications at 55 and 75 DAT in rice plants is presented in Table 3. The photosynthesis rates of plants treated with the Si topdressed and Si foliar spray methods were significantly different at 55 DAT, with the values of 11.3 and 11.0 *μ*mol CO_2_ m^−2^ s^−1^, respectively. Moreover, both Si treatments increased the Pn by 30 (Si foliar applied methods) and 26% (Si topdressed methods).

Stomatal conductance measurements shared similarities with the photosynthesis rate (as shown in Table 3). Plants treated with Si topdressing treatments were significantly different in stomatal conductance at 55 DAT, with a value of 183 mmol H_2_O m^−2^ s^−1^, or by a 65% increase compared to other treatments.

The relative chlorophyll content of different Si method applications at 55 and 75 DAT in rice plants is shown in Table 3. Si foliar-treated plants increased in chlorophyll content, with the highest mean value of 32.52 SPAD unit at 55 DAT compared to the control. Likewise, the relative chlorophyll content showed no significant difference at 75 DAT.

### 3.3. Biomass Partitioning

**Figure 1:**
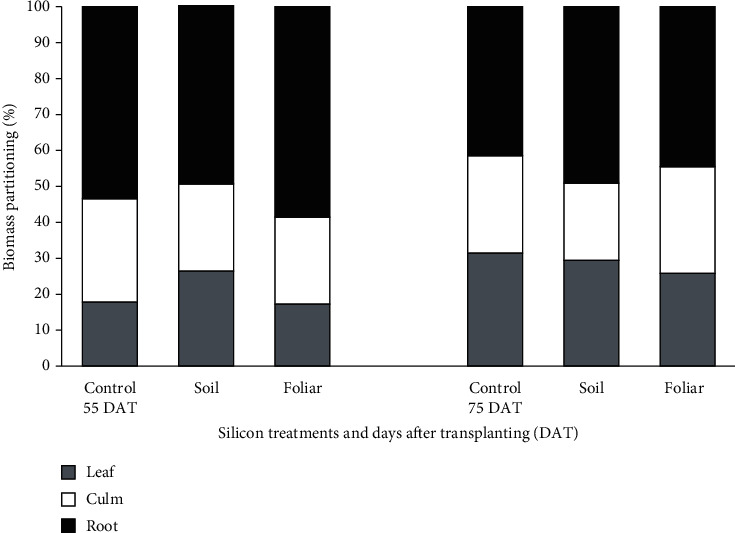
Whole plant percentage dry matter partitioning from different Si method applications at 55 and 75 DAT in rice plants.

Figure 1 and Table 4 illustrate the pattern of biomass partitioning in PadiU Putra rice plants with different Si method applications at 55 and 75 DAT. At 55 DAT, a statistical analysis revealed that Si foliar applied and Si topdressing treatments resulted in biomass partitioning to the leaves, culm, and roots being significantly different compared to the control (Table 4). Remarkably, Si foliar applied treatments were significantly different in biomass partitioning to the leaves, culm, and roots, following samples at 75 DAT. As for the total biomass partitioning, plants treated with Si foliar spray showed the highest dry matter production per hill at 55 and 75 DAT, where 43.4 and 63.2 g hill^−1^ were observed. Similar to the results from the root to shoot ratio, the application of Si foliar applied treatments showed the highest value compared to other treatments, with 1.41. Meanwhile, the application of Si topdressing treatments had the highest root to shoot ratio at 75 DAT, with 0.97.

In whole plant percentage dry matter partitioning, the highest dry matter partitioning occurred with the leaves, by 27%, in the Si topdressing; culm, by 29% in the control; and roots, by 59% in the Si foliar applied treatments. These were observed at 55 DAT (Figure 1). In contrast, biomass was partitioned to the leaves by 31% in the control; culm by 29% in the Si foliar applied, and roots by 49% in the Si topdressing treatments. These were observed at 75 DAT. The leaves and roots were most affected by the partitioning changes under both Si applied treatments, respectively, compared to other parts of the plant, especially at 55 DAT. In addition, at 75 DAT, Si foliar applied and Si topdressing treatments affected greater culm and root development of the rice plants compared to the control. According to the biomass partitioning, Si topdressing treatments improved dry matter partitioning into the leaves by 9% and 8% at 55 and 75 DAT, respectively, compared to the control. A similar pattern was observed in the Si foliar applied treatments, where 5% and 3% of dry matter partitioning into roots and culm, respectively, were enhanced, compared to the control treatments.

### 3.4. Crop Growth Map Using Unmanned Aerial Vehicle (UAV) and RGB Digital Camera

**Figure 2:**
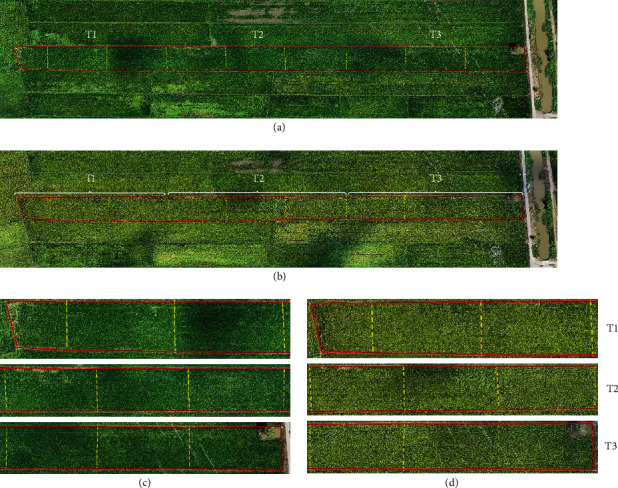
Boundary of the study area in this research at Tunjang, Jitra, Kedah, Malaysia at (a) 55 and (b) 75 DAT. Boundary of each of the treatment plots (T1: control/untreated plants; T2: Si topdressing plants; and T3: Si foliar applied plants) at (c) 55 and (d) 75 DAT.

Figure 2 shows the boundary of the study area in this research during the main planting season, made using a multirotor UAV at an altitude of 80 metres from the ground to monitor rice crop management and yield prediction, at 55 and 75 DAT. The red, green, and blue (RGB) image illustrates that the whole of the study area was about 1.42 ha. Based on Figures 2(a)–2(d), the rice plants showed uniformly as a dark green colour in the plot, indicating better plant growth performance at 55 DAT, especially from foliar applied treatments. Similar findings were observed at 75 DAT, where the rice plants consistently demonstrated a light yellowish colour in all plots. However, in the control treatment, darker greenish and yellow colours were spotted around the plot.

### 3.5. Scanning Electron Microscopy (SEM) Observations

#### 3.5.1. SEM Investigation of Rice Leaves at 55 and 75 DAT

**Figure 3:**
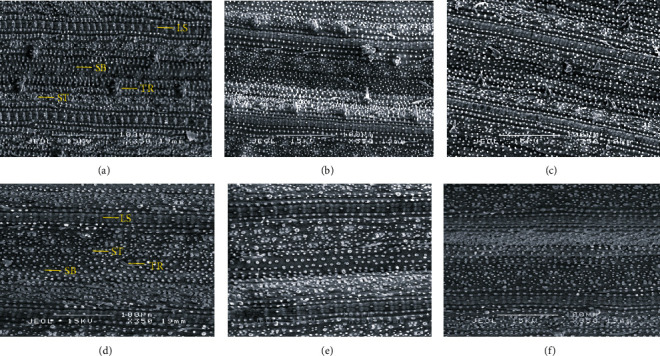
SEM image of the epidermal region of a rice plant leaf. The white granular areas of Si were detected on the abaxial leaf surfaces (a–c) at 55 DAT and (d–f) at 75 DAT at magnification 350x. LS: ladder-like structures; SB: silica bodies; TR: trichomes; ST: stomas.

In SEM observations, various structures were identified in the rice leaves, including silica bodies, ladder-like structures or dumbbell-shaped trichomes (fine outgrowths or appendages on plants), and stomas (as shown in Figure 3). Essentially, identical structures were found on all treatments of the leaves, regardless of the sampling times.

Si foliar applied treatments were significantly different in terms of the number of trichomes per field of view at 75 DAT (as shown in Table 5). Trichome distribution in Si foliar applied treatments was the highest by 29%, compared to other treatments. Similar findings were noted with the length of the ladder-like structures. The Si topdressing and Si foliar applied treatments were significantly different to the control at 75 DAT, with 9.95 and 9.97 *μ*m, respectively. The leaf samples of plants treated with both Si treatments showed a 13% extralength ladder-like structure in a row of silica cells and small silica bodies all around the sample.

The adaxial surfaces of the plants with Si topdressing treatments showed the highest number of silica bodies per view with 248 (18%) and 262 (24%), respectively, at both sampling times (Table 6). However, Si foliar applied treatments and control treatments were slightly different in terms of the number of silica bodies per view, observed at 55 and 75 DAT. In another parameter, the distance between the adjacent silica bodies was significantly different in Si topdressing treatments (5.40 *μ*m) at 55 DAT, compared to other treatments. Interestingly, plants applied with Si topdressing and Si foliar spray at 75 DAT showed significantly higher distances between adjacent silica bodies than the control; the former two treatments showed 6.37 and 6.32 *μ*m, respectively.

Table 7 shows the stomatal characteristics of rice leaves with different Si method applications at 55 and 75 DAT in rice plants. The highest number of stomata per field of view was obtained when Si was applied as foliar spray and for Si topdressed plants at 55 and 75 DAT, respectively. Si foliar applied and Si topdressing treatments showed an increased number of stomata per view, by 50 and 19% at 55 and 75 DAT, respectively. As for the method of application, the stomata diameters of Si foliar applied and Si topdressing were significantly higher than the control at 75 DAT, at 17.40 *μ*m. Similar results were found with stomatal width, where Si foliar applied (2.48 *μ*m) and Si topdressing (2.36 *μ*m) were significantly different than the control at 55 DAT. Remarkably, both Si treatments improved stomata diameter by 117% and stomata width by 39% in Si foliar applied treatments at 75 DAT. Si foliar applied at 75 DAT had the highest stomatal width, followed by Si topdressing treatments and the control, though they were insignificantly different. Stomatal width in rice plant leaves had a markedly increased photosynthesis rate in this study using the Si treatments. There was a positive linear relationship between stomatal width and the photosynthesis rate for all treatments, with correlation *y* = 0.2948*x* − 0.4951. The significant coefficients of 0.85 in the relationship showed that the highest stomatal width was obtained with a 5.63 *μ*m photosynthesis rate in Si foliar applied treatments at 75 DAT.

#### 3.5.2. SEM Investigation in Rice Culm at 55 and 75 DAT

**Figure 4:**
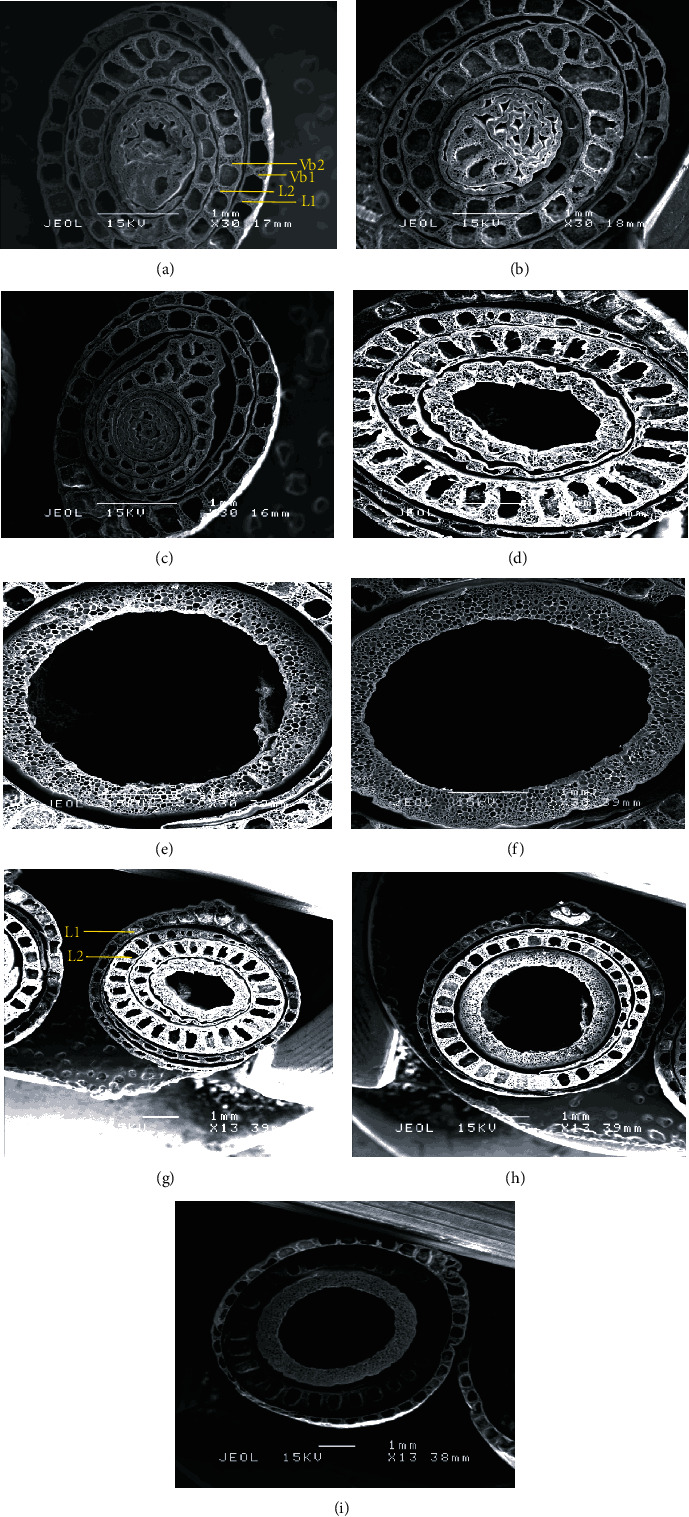
SEM image of the epidermal regions of rice plant culm sheaths. (a–c) at 55 DAT, (d–f) at 75 DAT at magnification 30x, and (g, h) at 75 DAT at magnification 13x. vb: vascular bundles.

To further identify the factors responsible for the effects of different Si method applications on the rice plant strength, the anatomical structure of the plants was analysed for each of the treatments with ×30 and ×200 magnification, as shown in Figure 4. In the transverse sections, the characteristics of layer thickness and vascular bundles were observed.

The diameter of the culm was significantly different in both Si method applications (Table 8). The diameter of the culm of the topdressing treatments (4171 mm) was significantly higher than the Si foliar applied treatments and control plants at 55 DAT. Interestingly, similar findings relating to the width and middle width of the culm were obtained in Si topdressing treatments at 55 DAT, with 2888 and 2147 mm, respectively. The diameter and width of the culm were higher when Si was applied as topdressing; they increased by 33% and 9%, respectively, compared to the Si foliar applied treatments and the control. Like the middle with of the culm, Si topdressing treatments showed the broadest rice culm at 31%.

The culm wall thickness of the first layer was significantly different depending on the method of Si application (Table 9). Si applied as topdressing treatments had the highest culm wall thickness of the first layer, followed by the Si foliar treatments and the control at 55 DAT. Meanwhile, Si foliar applied treatments were significantly higher than other treatments at 75 DAT. Likewise, the culm wall thickness of the second layer showed no significance at 55 and 75 DAT. In the Si topdressing and Si foliar applied treatments, the culm wall thickness of the first layer was the highest, with 9 and 24% at 55 and 75 DAT, respectively. The culm wall thickness of the third layer was significantly different for the Si foliar applied treatments, with 387 *μ*m at 55 DAT (24%). The equation correlation between the culm wall thickness and the height of the plant was *y* = −1.9362*x* + 461.79 with *R*^2^ = 0.5193. There was a negative linear relationship between them. The negative correlation indicated that the thickness of the culm increased, while the height of the plant decreased.

**Figure 5:**
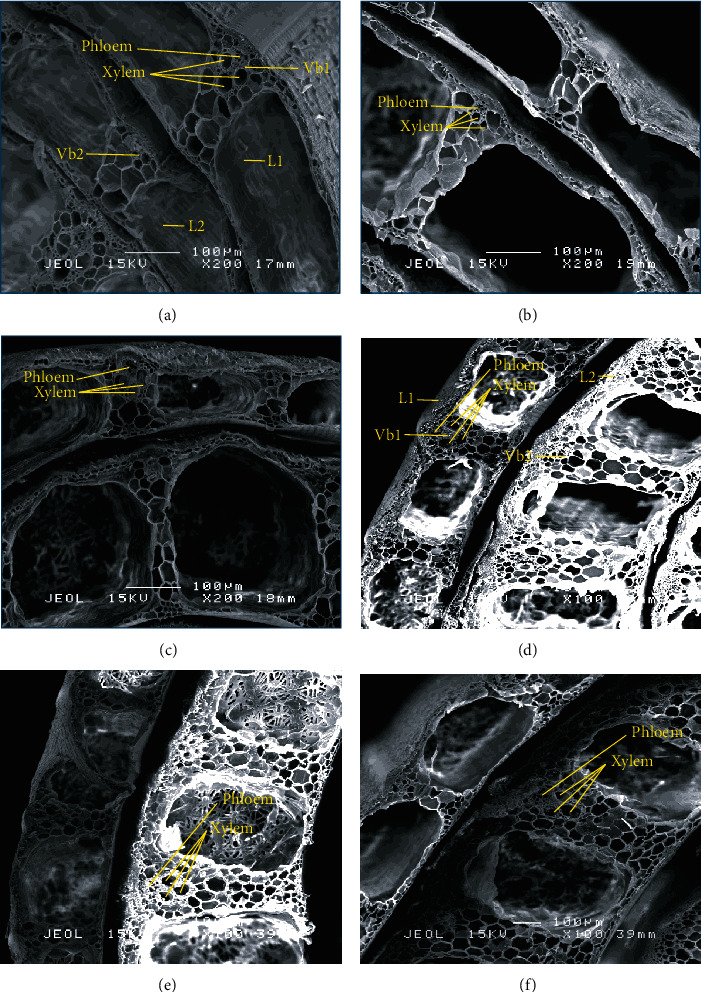
SEM image of the epidermal regions of rice plants. (a–c) At 55 DAT and (d–f) at 75 DAT at magnification 200x. L1: first layer; L2: second layer; Vb1: vascular bundle at first layer; Vb2: vascular bundle at second layer.

The images and results showed the presence of vascular bundles in the culm crosssections of rice plants in the outer and inner layers with 200x of magnification, regardless of treatment, are shown in Figure 5 and Table 10. However, there were differences in terms of the diameter and width of vascular bundles on both layers. In the control plants, the diameter and width of vascular bundles on the outer layers were widened more at early data collection (55 DAT) compared to other treatments. This was in contrast to Si foliar applied treatments, which showed significant differences in terms of diameter and width of the outer layer of vascular bundles and the inner layer of the diameter of vascular bundles with 161.5 (58%), 134.0 (181%), and 204.0 (80%) *μ*m at 75 DAT, respectively.

### 3.6. Yield Component at 120 DAT

The panicle length, number of grains per panicle, and percentage of filled grain were significantly different between treatments at 120 DAT (as shown in Table 11). Following that, Si foliar applied and Si topdressing treatments had the highest panicle length, with 26.67 and 26.53 cm, respectively. Meanwhile, the number of grains per panicle was increased in Si foliar applied treatments by 14% compared to the control. Similar results were obtained with the grain filling percentage in Si foliar applied treatments, where an increase of 95.2% was obtained. On the other hand, Si topdressing treatments improved by 8% in terms of thousand-grain weight compared to other treatments. Interestingly, the yield production of rice significantly increased in Si foliar applied, followed by the Si topdressing treatments, by 53 and 39%, respectively, compared to the control. A relatively higher rice yield was associated with the Si foliar applied and Si topdressing treatments, with 6.8 and 6.15 t ha^−1^, compared to the national Malaysia average yield of 4.8 t ha^−1^.

### 3.7. Economic Viability of Adopting Si Topdressing and Si Foliar Applied Treatments in Rice Plants

**Figure 6:**
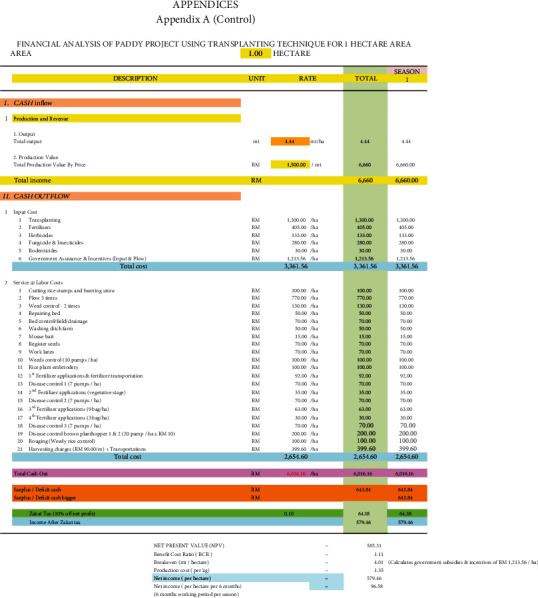
Financial analysis of paddy project using transplanting technique for one hectare of area (control).

**Figure 7:**
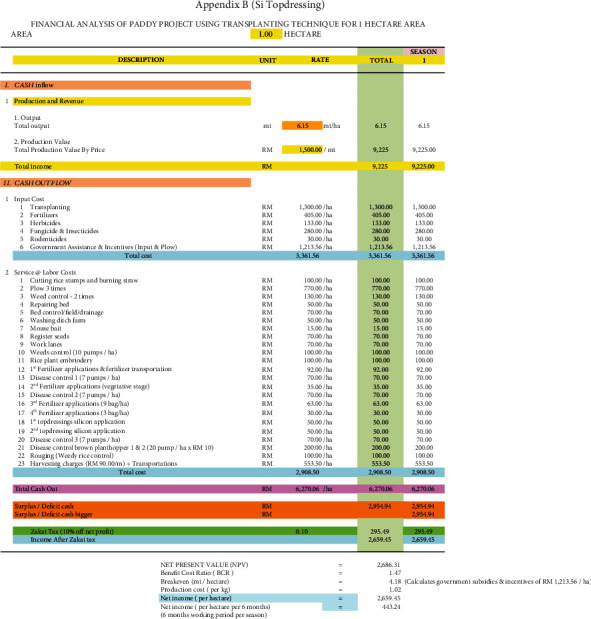
Financial analysis of paddy project using transplanting technique for one hectare of area (Si topdressing).

**Figure 8:**
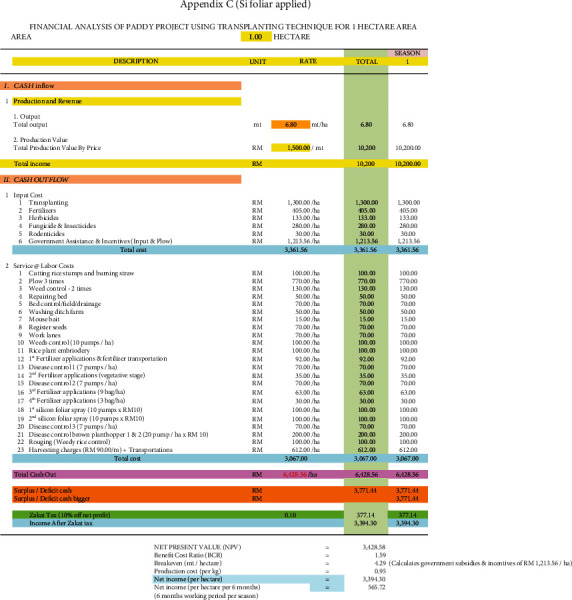
Financial analysis of paddy project using transplanting technique for one hectare of area (Si foliar applied).

Table 12 summarises the benefit-cost ratio (BCR) and net present value (NPV) of the different Si treatments used in rice cultivation with the Padiu Putra rice variety (Figures 6–8). The Si foliar applied and Si topdressing treatments produced higher yield and were more profitable during this planting season compared to the control. From an economic perspective (BCR), for every RM 1 spent in adopting the Si foliar applied and Si topdressing treatments, RM 1.59 and RM 1.47 were gained in return, respectively. In terms of NPV, the Si foliar applied and Si topdressing treatments were more economically viable compared to the control, producing an additional income of RM 2843.27 ha^−1^ and RM 2101 ha^−1^, respectively. The income generation of farmers improved by 486 and 359% with the Si foliar applied and Si topdressing treatments.

## 4. Discussion

Rice lodging is associated with height reduction, as stems weaken when supporting the weight of the grain, causing it to fall over. This is becoming a significant factor in rice production and often results in significant yield losses. Si application treatment is one of the approaches to fortify the leaf and culm sheaths and ultimately enhance rice yield. Broadcasting techniques, such as topdressing and foliar spray, are the common fertilisers used by the farmers, as they cost less but are very effective.

Reducing the plant height and internode length and the diameter of the internode are the first responsive events of plants to lodging and commonly regulated by Si amendments [34]. There were positive indications with Si application in terms of physiological measurement such as photosynthesis, chlorophyll content [12, 35], and biomass partitioning [36]. These physiological measurements were supported with images using the UAV and can be used for rice yield predictions [30, 37].

Si foliar applied treatments that are capable of altering physiological and anatomical structures are preferred, for they could fortify the leaf and culm sheaths and ultimately increase rice yield production. The greater improvement of biomass partitioning to the leaf and root in the early stages using Si topdressing and foliar applied was followed by the culm and root being affected at the reproductive stages by both Si treatments, respectively. It also improved the biomass partitioning, mostly to the culm and root, through improvements in photosynthetic efficiency [38]. The assimilation rates in photosynthetic leaves increased due to enhanced photosynthetic metabolites and enzyme activity, which directly improved grain production. The ratio of dry matter accumulation in roots to shoots with Si foliar applied treatments was greater than in the control plants, which clearly indicated the altered partitioning of resources within the rice plants. This might be due to the higher translocation of nutrients in roots, which influenced the movement of ions from the soil to the plant leaves. Similar results were reported by [39–41].

Using scanning electron microscopy (SEM), an extralong ladder-like structure and an intense number of silica bodies on the leaf structures for both Si treatments were observed. Silica content could be well-deposited in the leaf epidermis of rice plants and form a thick layer as a support structure and for the protection of the leaf [42, 43]. It was assumed that the consistently intense lignin accumulation in the leaf was for self-defence mechanism purposes; similar findings were observed by [12]. Interestingly, Si foliar applied treatments might alter the leaves stomatal width, thereby enhancing the stomatal aperture and improving photosynthetic efficiency for better dry matter accumulation [40, 41].

The strengthened culm sheath structures in Si-treated plants might be due to the thickness of the cell walls in the sclerenchyma and more vascular bundles in both the peripheral and the inner sections of the outer layers [44]. Similar findings with both Si treatments were found according to [12, 45]. That indicates an efficient assimilated translocation process from root to shoot in Si treatments.

Greater yield production with Si treatment showed that rice plants improve their photosynthesis and assimilated distribution [46, 47]. Spikelets per panicle and grain filling percentage were higher when Si was applied as foliar spray, compared to the other treatments. Compared to this parameter, the percentage of filled grain could potentially reveal more about the effects of Si supplementation as it is an indicator of sterility and fertility. Therefore, yield seems to be maximised when Si was supplied as foliar spray at the reproductive stage, which is also referred to as preheading [10]. Interestingly, BCR and NPV showed the better economic viability of adopting Si foliar applied treatments in rice plants. These results suggest the ability of Si foliar applied treatments to enhance farmers' income generation tremendously [47–53]. Thus, the Si foliar spray is the most suitable method of application and could be incorporated in combination with fungicides and insecticides to aid the supply of nutrients to crops in the form of sprays [10].

Further study should be conducted on the mechanism of Si foliar applied treatments on the plant uptake through the leaf and root; the enzymes and hormonal regulation related to yield improvement, such as sucrose synthase enzyme; and the molecular identification of genes associated with hormonal regulation.

## 5. Conclusions

Si foliar applied treatments affect the plant growth, physiological process, leaf and culm morphology, and the yield of rice. The adjustment of the leaves' stomatal width, the enhancement of the stomatal aperture, the more vascular bundles in the culm, and the efficient photosynthesis rate lead to better dry matter accumulation from root to shoot with Si foliar applied treatments. Therefore, rice yield production increased tremendously with a higher benefit-cost ratio and net present value.

## Figures and Tables

**Table 1:** Plant height and tiller number on different Si method applications at 55 and 75 DAT in rice plants.

Treatment	Plant height (cm)		Tiller number (m^−2^)	
	DAT			
	55	75	55	75
Control	79.7a	84.5a	438a	513a
Topdressing	66.3b	83.0a	429a	460a
Foliar spray	69.3b	81.3a	379a	432a
LSD _(__*P*=0.05__)_	6.84	4.55	17.05	17.59
CV	4.21	2.42	4.99	12.00

Mean values followed by the same letters within a column are not significantly different at *P* ≤ 0.05 by the LSD test. DAT: days after transplanting; CV: coefficient of variation.

**Table 2:** Diameter and length of internodes on different Si method applications at 55 and 75 DAT in rice plants.

Treatment	Diameter of internodes at 20 cm (mm)		Length of internodes (mm)	
	DAT			
	55	75	55	75
Control	2.72a	4.9a	35.83b	63.74a
Topdressing	2.66a	3.5c	43.85a	55.99b
Foliar spray	2.04a	4.0b	30.51c	58.27b
LSD _(__*P*=0.05__)_	0.75	0.45	5.38	3.52
CV	13.31	4.73	7.73	2.62

Mean values followed by the same letters within a column are not significantly different at *P* ≤ 0.05 by the LSD test. DAT: days after transplanting; CV: coefficient of variation.

**Table 3:** Photosynthesis rate, stomatal conductance, and relative chlorophyll content from different Si method applications at 55 and 75 DAT in rice plants.

Treatment	Photosynthesis rate (*μ*mol CO_2_ m^−2^ s^−1^)		Stomatal conductance (mmol H_2_O m^−2^ s^−1^)		Relative chlorophyll content (SPAD value)	
	DAT					
	55	75	55	75	55	75
Control	8.7b	17.8a	111.0b	314.7a	29.5b	34.6a
Topdressing	11.3a	16.2b	183.0a	269.0a	26.6b	31.2a
Foliar spray	11.0a	15.8b	102.9b	218.5a	32.5a	35.6a
LSD _(__*P*=0.05__)_	2.03	1.34	12.58	18.16	5.23	3.53
CV	8.69	3.55	4.19	12.89	3.61	4.61

Mean values followed by the same letters within a column are not significantly different at *P* ≤ 0.05 by the LSD test. DAT: days after transplanting; CV: coefficient of variation.

**Table 4:** Dry matter production on different Si method applications at 55 and 75 DAT in rice plants.

Biomass partitioning	Leaf (g hill^−1^)		Culm (g hill^−1^)		Root (g hill^−1^)		Total (g hill^−1^)		Root : shoot ratio	
DAT	55	75	55	75	55	75	55	75	55	75
Control	2.9b	16.9a	4.7b	14.6b	8.7c	22.3b	16.3c	53.8b	0.49c	0.72c
Topdressing	6.4a	14.4b	5.9b	10.5c	12.0b	24.0b	24.2b	48.9b	0.98b	0.97a
Foliar spray	7.5a	16.3a	10.5a	18.8a	25.4a	28.1a	43.4a	63.2a	1.41a	0.80b
LSD _(__*P*=0.05__)_	0.57	2.12	1.88	1.99	0.69	3.77	1.37	0.69	3.33	2.12
CV	7.98	8.62	5.67	7.65	7.64	6.03	10.09	7.46	5.64	3.49

Mean values followed by the same letters within a column are not significantly different at *P* ≤ 0.05 by the LSD test. DAT: days after transplanting; CV: coefficient of variation.

**Table 5:** Number of trichomes, ladder-like structures, and length of ladder-like structures of rice leaves with different Si method applications at 55 and 75 DAT.

Treatment	Number of trichomes per field of view (FOV)		Number of ladder-like structure per field of view (FOV)		Length of ladder-like structure (*μ*m)	
	DAT					
	55	75	55	75	55	75
Control	6a	5b	26a	15a	5.75a	8.67b
Topdressing	5a	4b	26a	16a	4.09b	9.95a
Foliar spray	8a	7a	24a	14a	3.49b	9.97a
LSD _(__*P*=0.05__)_	4.16	1.15	3.83	1.73	1.58	2.87
CV	2.45	12.50	2.79	6.39	4.25	7.39

Mean values followed by the same letters within a column are not significantly different at *P* ≤ 0.05 by the LSD test. DAT: days after transplanting; CV: coefficient of variation. Number of trichomes and ladder-like structures (FOV) were counted at magnification 350x.

**Table 6:** Characteristics of rice leaves in terms of number of silica bodies and distances between silica bodies from different Si method applications at 55 and 75 DAT in rice plants.

Treatment	Number of silica bodies per field of view (FOV)		Distance between adjacent silica bodies (*μ*m)	
	DAT			
	55	75	55	75
Control	211b	211b	4.49b	5.22b
Topdressing	248a	262a	5.40a	6.37a
Foliar spray	211b	216b	4.36b	6.32a
LSD _(__*P*=0.05__)_	12.93	11.96	1.12	1.77
CV	7.89	2.90	6,40	1.97

Mean values followed by the same letters within a column are not significantly different at *P* ≤ 0.05 by the LSD test. DAT: days after transplanting; CV: coefficient of variation. Number of silica bodies (FOV) was counted at magnification 1000x.

**Table 7:** Stomatal characteristics of rice leaves with different Si method applications at 55 and 75 DAT.

Treatment	Number of stomata per field of view (FOV)		Stomatal diameter (*μ*m)		Stomatal width (*μ*m)	
	DAT					
	55	75	55	75	55	75
Control	4b	46b	12.00a	8.02b	2.06b	4.06b
Topdressing	5b	57a	12.18a	17.40a	2.36a	4.08b
Foliar spray	8a	47b	10.80a	17.40a	2.48a	5.63a
LSD _(__*P*=0.05__)_	2.11	11.96	1.81	6.06	1.79	1.53
CV	8.63	2.40	5.50	8.32	5.19	9.10

Mean values followed by the same letters within a column are not significantly different at *P* ≤ 0.05 by the LSD test. DAT: days after transplanting; CV: coefficient of variation. Number of stomata (FOV) was counted at magnification 1000x.

**Table 8:** The diameter and width of the culm with different Si method applications at 55 and 75 DAT in rice plants.

Treatment	Culm diameter (mm)		Culm width (mm)		Culm middle diameter (mm)		Culm middle width (mm)	
	DAT							
	55	75	55	75	55	75	55	75
Control	3130b	7927a	2639b	4888a	2254a	3678a	1782a	1942b
Topdressing	4171a	5684b	2888a	3981b	2147a	2782b	1571a	2543a
Foliar spray	3307b	7310a	2146c	4218a	1653b	3216a	1261a	767c
LSD _(__*P*=0.05__)_	5.64	7.40	4.99	5.70	3.14	6.80	13.21	12.00
CV	6.50	9.19	6.82	5.67	8.37	8.90	6.92	5.62

Mean values followed by the same letters within a column are not significantly different at *P* ≤ 0.05 by the LSD test. DAT: days after transplanting; CV: coefficient of variation.

**Table 9:** Culm wall thickness of rice plants with different Si method applications at 55 and 75 DAT.

Treatment	Culm wall thickness of the 1^st^ layer (*μ*m)		Culm wall thickness of the 2^nd^ layer (*μ*m)		Culm wall thickness of the 3^rd^ layer (*μ*m)	
	DAT					
	55	75	55	75	55	75
Control	308b	266c	322a	305a	312b	339a
Topdressing	337a	295b	338a	293a	295b	320a
Foliar spray	294b	330a	324a	286a	387a	344a
LSD _(__*P*=0.05__)_	10.7	18.01	10.5	14.87	12.73	15.04
CV	2.45	6.30	7.78	3.45	7.50	3.98

Mean values followed by the same letters within a column are not significantly different at *P* ≤ 0.05 by the LSD test. DAT: days after transplanting; CV: coefficient of variation.

**Table 10:** Vascular bundle characteristics of rice culm with different Si method applications at 55 and 75 DAT.

Treatment	Outer layer diameter of vascular bundles (*μ*m)		Outer layer width of vascular bundles (*μ*m)		Inner layer diameter of vascular bundles (*μ*m)		Inner layer width of vascular bundles (*μ*m)	
	DAT							
	55	75	55	75	55	75	55	75
Control	79.8a	102.0b	58.8a	47.7c	59.5a	113.6b	48.8a	121.0a
Topdressing	53.8b	96.9b	26.7b	102.0b	77.8a	107.1b	49.4a	100.2b
Foliar spray	60.4b	161.5a	27.8b	134.0a	70.9a	204.0a	40.3a	117.0ab
LSD _(__*P*=0.05__)_	17.88	25.95	18.98	4.61	19.48	25.53	32.28	17.16
CV	7.95	9.55	6.98	9.76	7.80	5.76	8.80	7.91

Mean values followed by the same letters within a column are not significantly different at *P* ≤ 0.05 by the LSD test. DAT: days after transplanting; CV: coefficient of variation.

**Table 11:** Yield components of rice plants at 120 DAT with different Si method applications at 55 and 75 DAT.

Treatment	Panicle length (cm)	Spikelets per panicle	Filled grain (%)	1,000-grain weight (g)	Yield (tha^−1^)
Control	24.77b	175b	89.53b	26.03b	4.44c
Topdressing	26.53a	175b	91.87b	28.13a	6.15b
Foliar spray	26.67a	200a	95.20a	26.33b	6.80a
LSD _(__*P*=0.05__)_	3.33	5.88	8.52	2.12	6.84
CV	5.64	8.63	4.08	3.49	4.21

Mean values followed by the same letters within a column are not significantly different at *P* ≤ 0.05 by the LSD test. CV: coefficient of variation.

**Table 12:** Effects of different Si method applications on benefit-cost ratio (BCR) and net present value (NPV) in rice plants at 120 DAT.

Treatment	Benefit-cost ratio	Net present value (RM ha^−1^)
Control	1.09c	558.04c
Topdressing	1.49b	2749.95b
Foliar spray	1.63a	3583.13a
LSD _(__*P*=0.05__)_	1.88	1.55
CV	5.67	2.45

Mean values followed by the same letters within a column are not significantly different at *P* ≤ 0.05 by the LSD test. CV: coefficient of variation.

## Data Availability

Regarding on data availability, we would like to declare that all data related to the work provided in the manuscript are submitted.

## References

[1] Sheehy J. E., Mitchell P. L. (2011). *Rice and global food security: the race between scientific discovery and catastrophe. ACCESS NOT EXCESS—the search for better nutrition*.

[2] Ministry of Agriculture and Agro-based Industries, Malaysia (2013). *Paddy Statistics of Malaysia*.

[3] Ismail S. (2017). Dasar dan polisi industri padi dan beras (Rice and rice industry policies and policies). http://padi.mardi.gov.my/dokumen/slide/L1.

[4] Miah G., Rafii M. Y., Ismail M. R. (2015). Inheritance patterns and identification of microsatellite markers linked to the rice blast resistance in BC_2_F_1_ population of rice breeding. *Bragantia*.

[5] Tanweer F., Rafii M. Y., Sijam K. (2015). Introgression of blast resistance genes (putative Pi-b and Pi-kh) into elite rice cultivar MR219 through marker-assisted selection. *Frontiers in Plant Science*.

[6] Fageria N. K., Baligar V. C., Ralph C. (2006). *Physiology of Crop Production*.

[7] Gowariker V., Krishnamurthy V. N., Gowariker S., Dhanokar M., Paranjape K. (2009). *The Fertilizer Encyclopedia*.

[8] Zhang J., Li G., Song Y. (2014). Lodging resistance characteristics of high-yielding rice populations. *Field Crops Res.*.

[9] Cuong T. X., Ullah H., Datta A., Hanh T. C. (2017). Effects of silicon-based fertilizer on growth, yield and nutrient uptake of rice in tropical zone of Vietnam. *Rice Science*.

[10] Dorairaj D., Ismail M. R., Sinniah U. M., Tan K. B. (2020). Silicon mediated improvement in agronomic traits, physiological parameters and fiber content in *Oryza sativa*. *Acta Physiol. Plant*.

[11] Zhang C., Wang L., Zhang W., Zhang F. (2013). Do lignification and silicification of the cell wall precede silicon deposition in the silica cell of the rice (*Oryza sativa* L.) leaf epidermis?. *Plant and Soil*.

[12] Dorairaj D., Ismail M. R., Sinniah U. R., Tan K. B. (2017). Influence of silicon on growth, yield and lodging resistance of MR219, a lowland rice of Malaysia. *Journal of Plant Nutrition*.

[13] Keegstra K. (2010). Plant cell walls. *Plant Physiology*.

[14] Sposito G. (1989). *The Chemistry of Soils*.

[15] Rodrigues F. Á., Jurick W. M., Datnoff L. E., Jones J. B., Rollins J. A. (2005). Silicon influences cytological and molecular events in compatible and incompatible rice- _Magnaporthe grisea_ interactions. *Physiological and Molecular Plant Pathology*.

[16] Epstein E. (1994). The anomaly of silicon in plant biology. *Proceedings. National Academy of Sciences. United States of America*.

[17] Mitani N., Ma J. F. (2005). Uptake system of silicon in different plant species. *Journal of Experimental Botany*.

[18] Lavinsky A. O., Detmann K. C., Reis J. V. (2016). Silicon improves rice grain yield and photosynthesis specifically when supplied during the reproductive growth stage. *Journal of Plant Physiology*.

[19] Mo Z. W., Lei S., Ashraf U. (2017). Silicon fertilization modulates 2-acetyl-1-pyrroline content, yield formation and grain quality of aromatic rice. *Journal of Cereal Science*.

[20] Cai Y., Zhang S., Cai K., Huang F., Pan B., Wang W. (2020). Cd accumulation, biomass and yield of rice are varied with silicon application at different growth phases under high concentration cadmium-contaminated soil. *Chemosphere*.

[21] Ma J. F., Takahashi E. (2002). Silicon uptake and accumulation in plants. *Soil, Fertilizer, and Plant Silicon Research in Japan*.

[22] Farnaz A.-A., Jugah K., Ahmad S., Ahmad Husni M. H., Abbas N. (2012). Effect of foliar and root application of silicon against rice blast fungus in MR219 rice variety. *Plant Pathol. J*.

[23] Gao M., Zhou Z., Liu H. L. (2018). Foliar spraying with silicon and selenium reduces cadmium uptake and mitigates cadmium toxicity in rice. *Sci. Total Environ.*.

[24] Gan X. Q., Jiang L. G., Xu J. Y., Dong D. F., Wei S. Q. (2004). Characteristics and genotypic difference of silicon accumulation and distribution in rice. *Plant Nutrition and Fertitizer Science*.

[25] Clarke R. (2014). Understanding the drone epidemic. *Computer Law and Security Review*.

[26] Xue X. Y., Lan Y. B. (2013). Agricultural aviation applications in USA. *Transactions CSAM.*.

[27] Jones H. G., Vaughan R. A. (2010). *Remote Sensing of Vegetation: Principles, Techniques, and Applications*.

[28] Yoshida S. (1981). Physiological analysis of rice yield. *Fundamentals of Rice Crop Science*.

[29] Hunt R. (1978). *Plant Growth Analysis*.

[30] Norasma N. C. Y. (2016). *Site-specific weed management using remote sensing. [PhD Thesis]*.

[31] Ibrahim A. Z., Siwar C. (2012). *Kawasan Pengairan Muda: Merentasi Masa Menyangga Keselamatan Makanan Negara*.

[32] Nwaobiala C. U., Adesope O. M. (2013). Economic analysis of small holder rice production systems in Ebonyi State South East, Nigeria. *Russian Journal of Agricultural and Socio-Economic Sciences*.

[33] Ingabire C., Bizoza A. R., Mutware J. (2013). Determinants and profitability of rice production in Cyabayaga Watershed, Eastern Province, Rwanda. *Rwanda Journal, Series H: Economics and Management.*.

[34] Feng Ma J., Yamaji N., Mitani-Ueno N. (2011). Transport of silicon from roots to panicles in plants. *Proceedings of the Japan Academy, Series B*.

[35] Amirul M. A., Abdul Shukor J., Rafii M. Y., Azizah A. H. (2015). Effect of salinity on biomass yield and physiological and stem-root anatomical characteristics of Purslane (Portulaca oleracea L.) accessions. *BioMed Research International*.

[36] Alcázar R., Altabella T., Marco F. (2011). Polyamines: molecules with regulatory functions in plant abiotic stress tolerance. *Planta*.

[37] Chauhan S., Darvishzadeh R., Boschetti M., Pepe M., Nelson A. (2019). Remote sensing-based crop lodging assessment: current status and perspectives. *J. Photogramm. Remote Sens.*.

[38] Guo J., Xu W., Yu X. (2016). Cuticular wax accumulation is associated with drought tolerance in wheat near-isogenic lines. *Frontiers in Plant Science*.

[39] Kim H.-Y., Lim S.-S., Kwak J.-H. (2011). Dry matter and nitrogen accumulation and partitioning in rice (*Oryza sativa* L.) exposed to experimental warming with elevated CO_2_. *Plant and Soil*.

[40] Puteh A., Mondal M. M. A. (2014). Salinity stress during booting and heading stages affects yield in rice. *Life Science Journal*.

[41] Zulkarami B., Deivaseeno D., Halimi M. S., Muhammad Husni O., Nazmin Y., Mohd Razi I. (2018). Water stress affects growth, biomass partitioning, grain filling and productivity of MR219, a high yielding rice variety of Malaysia. *Int. J. Agric. Environ. Bio-Res.*.

[42] Piperno D. R., Pearsall D. M. (1998). The Silica Bodies of Tropical AmericanGrasses: Morphology, Taxonomy, and Implications for Grass Systematics and Fossil Phytolith Identification. *Smithsonian Contributions to Botany*.

[43] Meunier J. D., Barboni D., Anwarul Haq M. (2017). Effect of phytoliths for mitigating water stress in durum wheat. *The New Phytologist*.

[44] Kim S. G., Kim K. W., Park E. W., Choi D. (2002). Silicon induced cell wall fortification of rice leaves: a possible cellular mechanism of enhanced host resistance to blast. *Phytopathology*.

[45] Chaturvedi G. S., Misra C. H., Singh O. N., Pandey C. B., Yadav V. P., Singh A. K., Ingram K. T. (1995). Physiological basis and screening for tolerance for flash flooding. *Rainfed Lowland Rice, Agricultural Research in High Risk Environment*.

[46] Counce P., Gravois K. (2006). Sucrose synthase activity as a potential indicator of high rice grain yield. *Crop Science*.

[47] Zulkarami B., Dorairaj D., Halimi Mohd S., Mohd Razi I. (2019). Regulation of sucrose synthase and its association with grain filling in spermine-treated rice plant under water deficit. *Journal of Plant Interactions*.

[48] Reynolds M., Foulkes J., Furbank R. (2012). Achieving yield gains in wheat. *Plant, Cell and Environment*.

[49] Ashraf M., Akram N. A., Al-Qurainy F., Foolad M. R. (2011). Drought tolerance: roles of organic osmolytes, growth regulators and mineral, nutrients. *Advances in Agronomy*.

[50] Mohapatra P. K., Panigrahi R., Turner N. C. (2011). Physiology of spikelet development on the rice panicle: is manipulation of apical dominance crucial for grain yield improvement?. *Advances in Agronomy*.

[51] Zulkarami B., Panhwar Q. A., Mohd Razi I., Halimi M. S., Mondal M. M. A. (2014). An alternative and quicker strategy to improve rice (*Oryza sativa*) yield through application of phytohormones. *Trans. Malaysian Soc. Plant Physiol.*.

[52] Fageria N. K., Barbosa F. M. P., Moreira A., Guimaraes C. M. (2009). Foliar fertilization of crop plants. *Journal of Plant Nutrition*.

[53] Girma K., Martin K. L., Freeman K. W. (2007). Determination of optimum rate and growth stage for Foliar‐Applied phosphorus in corn. *Communications in Soil Science and Plant Analysis*.

